# Cognitive effort and active inference

**DOI:** 10.1016/j.neuropsychologia.2023.108562

**Published:** 2023-06-06

**Authors:** Thomas Parr, Emma Holmes, Karl J. Friston, Giovanni Pezzulo

**Affiliations:** a Wellcome Centre for Human Neuroimaging, Queen Square Institute of Neurology, UK; b Institute of Cognitive Sciences and Technologies, National Research Council, Rome, Italy

**Keywords:** Cognitive effort, Mental effort, Active inference, Variational, Covert attention, Stroop effect

## Abstract

This paper aims to integrate some key constructs in the cognitive neuroscience of cognitive control and executive function by formalising the notion of cognitive (or mental) effort in terms of active inference. To do so, we call upon a task used in neuropsychology to assess impulse inhibition—a Stroop task. In this task, participants must suppress the impulse to read a colour word and instead report the colour of the text of the word. The Stroop task is characteristically effortful, and we unpack a theory of mental effort in which, to perform this task accurately, participants must overcome prior beliefs about how they would normally act. However, our interest here is not in overt action, but in covert (mental) action. Mental actions change our beliefs but have no (direct) effect on the outside world—much like deploying covert attention. This account of effort as mental action lets us generate multimodal (choice, reaction time, and electrophysiological) data of the sort we might expect from a human participant engaging in this task. We analyse how parameters determining cognitive effort influence simulated responses and demonstrate that—when provided only with performance data—these parameters can be recovered, provided they are within a certain range.

## Highlights

• This paper offers a formalisation of ‘cognitive effort’ under the active inference framework.
• Cognitive effort is formulated as a deviation from prior beliefs about mental (covert) action—i.e., effort is exerted to overcome a mental habit.
• A computational model of the Stroop task—a characteristically effortful task—is developed to illustrate this notion of effort.
• We demonstrate that it is possible to recover combinations of effort-related model parameters from simulated data.

## Graphical abstract

**Figure:**
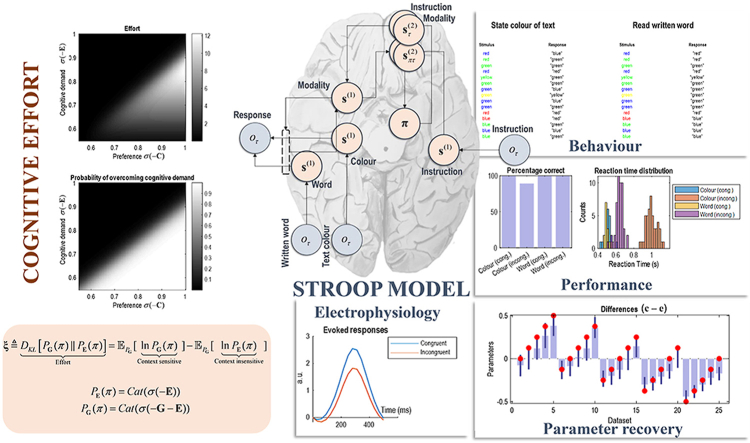

## 1 Introduction

What makes an activity effortful? A simple (and perhaps simplistic) answer is that effortful activities involve maintaining something in a state that it is not normally in. For instance, if you were to raise your arm and keep it in the air it will become progressively effortful to maintain this posture. However, it takes very little effort to keep your arm by your side—a posture that is much more common. This paper argues that this framing of effort is also applicable to cognitive, or mental, effort (terms we will use interchangeably). Notions of effort have long been considered in theories of attention. For example, Kahneman (1973) notes that ‘distraction is resisted at a cost.’ Here, we assume that the only difference between motor and cognitive effort is that the activity is covert as opposed to overt. This means we must appeal to the notion of a mental action (Limanowski and Friston, 2018; Pezzulo, 2018)—of which covert attention is perhaps the best-known example (Posner, 1980; Rizzolatti et al., 1987). To build some intuition as to what we are talking about, try to maintain visual fixation on the first word in this paragraph while reading the fourth word. It will have felt effortful to maintain fixation on the word ‘What’ and to resist looking at the word ‘activity’. As we will argue here, this represents the brain expending energy to violate a prior belief—here, that your focus of attention should be deployed such that it aligns with your fovea (and vice versa) (Manohar et al., 2015).

The implication of the above is that we need to consider prior beliefs about mental actions to understand effort. When these priors are formulated in terms of the kinds of behaviour we normally engage in, or behaviour we have engaged in frequently in the past, they can be thought of as mental (or cognitive) habits. This is not a new idea, and has been the focus of recent work that treats delusions as representing entrenched cognitive habits (Adams et al., 2021). However, it is a useful idea that lets us attempt to formulate cognitive effort in terms of violating a mental habit.^1^ The association between habits and priors provides a useful link with previous formulations of effort in information theoretic terms (Barceló and Cooper, 2018; Butz, 2022; Ortega and Braun, 2013; Zénon et al., 2019). These associate effort with redundancy, in the sense of efficient coding principles (Barlow, 1961), and with complexity (Sajid et al., 2020) in the Bayesian sense. In Bayesian statistics, complexity quantifies the degree to which we must update our prior beliefs to explain the data at hand (Jefferys and Berger, 1992). This must be offset against the accuracy with which we can predict those data in quantifying the fit of the model to those data. When actions come into play, they allow us to modify the data we will receive in the future, and so decisions about which action to take must be based upon anticipated data. If cognitive effort is analogous to a complexity cost, it can be formulated as the divergence between our habitual priors about our actions and our beliefs if we anticipate the consequences of acting in this particular context. In active inference, the context sensitive plausibility of an action is quantified using an expected free energy functional of allowable actions (Sajid et al., 2021a, b) that scores its salience or anticipated information gain (Lindley, 1956), under some prior preferences. We will see later how a formulation of mental planning—based upon expected free energy—leads naturally to a measure of cognitive effort.

Our approach here follows that of (Zénon et al., 2019), who frame effort explicitly in terms of complexity costs incurred through violating a prior belief about some ‘default’ policy. We build upon Zénon et al.‘s conceptual analysis with a complete quantitative model, capable of simulating behavioural and neronal responses. By formulating effort in terms of active inference, which rests upon probabilistic belief-updating, we work directly within an information-theoretic framework. This facilitates translation between the psychological concepts (like effort) and information theoretic quantities (like complexity). A key contribution of this formulation is that it enables one to recover the prior beliefs required to quantify complexity from behavioural data. Ultimately this may be important if the notion of effort is to be made practically useful in quantitative, empiricial cognitive, and perhaps clinical, research.

In the classic Stroop task (Stroop, 1935), participants are asked to report the font colour that a word is written in, while ignoring the semantics of the word. The word may be congruent with the font colour (e.g., the word ‘blue’ written in blue font), incongruent (e.g., the word ‘red’ written in blue font) or neutral (e.g., ‘xxxx’ written in blue font). In such tasks, the Stroop interference effect is the common finding that accuracy is worse, or reaction times are longer (or both), when the word is incongruent compared to neutral. The Stroop facilitation effect refers to improved accuracy (and or shorter reaction times) in congruent relative to neutral conditions. To keep things simple, we will consider only the congruent and incongruent conditions in this paper, such that the Stroop effect is a combination of facilitation and interference effects. We argue that the Stoop task is effortful because our normal mental habit is to read a word, and impulse inhibition requires us to overcome this mental habit when asked to report the font colour. In other words, an impulse represents the way in which we might normally respond to something—i.e., a mental plan that we commonly adopt—which can be considered a prior belief. Maintaining a belief that we should pursue an alternative plan therefore incurs a complexity cost that we experience as cognitive effort. Put simply, effort is the degree to which we have to ‘change our mind’ when updating prior beliefs about our behaviour, after considering the consequences of action in the context of task demands. Note that ‘beliefs’ in this setting are not propositional in nature, they refer to (subpersonal) Bayesian belief distributions that may or may not be accompanied by qualitative experience. The idea here is that ‘effort’ is the qualitative experience of committing to a behaviour that diverges from *a priori* habits.

There is a vast literature both on the Stroop task and on cognitive effort more generally (Altmann and Davidson, 2001; Chuderski and Smolen, 2016; Kalanthroff et al., 2018; Phaf et al., 1990; Verguts and Notebaert, 2009). While we cannot do this previous research justice in the space available in this article, it is worth briefly considering how our approach is situated relative to its predecessors. An influential computational model of the task was based upon a combination of a drift-diffusion model and a feedforward neural network (Cohen et al., 1990). The difference in behaviour between the word-reading and colour-naming conditions was elicited by providing more training on the former compared to the latter task. This was based upon the assumption that Stroop participants have more experience of reading words that naming their colours—an assumption we also adopt, but frame in terms of a prior belief. This style of modelling has been successful in reproducing a range of features of Stroop tasks, including increased reaction times when switching task (Gilbert and Shallice, 2002) and, with some modifications, features of functional imaging experiments (Herd, Banich and O'Reilly, 2006).

Our approach here offers a complementary perspective, which inverts the methods outlined above. Instead of attempting to connect artificial neural populations such that they can perform a task, we focus instead upon the structure of the task itself. This structure can be articulated in terms of a generative model and equipped with an objective function of the sort used in variational inference. By minimising the objective, we find an optimal solution to the task and attempt to identify neuronal dynamics, and a notion of mental effort, from this solution. A further advantage of this inferential perspective is that it provides a close link to information theoretic formulations of effort, as noted by Botvinick et al. (2001). However, it is also worth noting that the subtleties of Stroop task results have been much more thoroughly analysed in the papers cited above than we attempt in this paper, which simply uses the task to provide an illustration of the formulation on offer.

In what follows, we begin with a brief overview of the active inference formalism, with a focus on the importance of generative models. We then detail a generative model for the Stroop task and unpack its inversion; both in terms of behaviour and the electrophysiological manifestations of the requisite belief updating. Finally, we consider how maintaining different prior beliefs affects performance data, and whether we can infer parameters relating to cognitive effort from performance data alone.

## 2 Active inference and cognitive effort

The approach we adopt in this paper is based upon active inference (Parr et al., 2022). Active inference formulates behaviour and neuronal dynamics as resulting from updating prior beliefs (implicitly) held by the brain about the way in which sensory data are generated. From this perspective, differences in behavioural and neuronal responses among people can be characterised in terms of differences in prior beliefs that either reflect healthy variation among the population, or damage to brain structures in neurological disease (Adams et al., 2016; Mirza et al., 2018; Schwartenbeck and Friston, 2016). Our subsequent analysis of cognitive effort rests upon this form of computational neuropsychology, in which we can ask how differences in prior beliefs affect the performance of a neuropsychological task. To make this more explicit, we first provide a brief overview of active inference, with a focus on the association between prior beliefs and computational anatomy.

Active inference is a normative approach, which means it appeals to an optimality criterion. The measure of optimality is known in physics as negative (variational) free energy (Beal, 2003) and in machine learning as the evidence lower bound (or ELBO). The ELBO approximates Bayesian model evidence—which measures the fit between some generative model and the data we are hoping to explain. Assuming the brain makes use of a model to explain its sensations, we can formulate perceptual dynamics as maximising the ELBO to better approximate evidence, and action as sampling sensory data to ensure it better fits our model. In short, both action and perception work to maximise the ELBO or, equivalently, to minimise free energy:

(1) $$\begin{array}{l} u\leftarrow \underset{u}{\mathrm{argmin}}F\left(o\left(u\right),Q\right)\\ Q\leftarrow \underset{Q}{\mathrm{argmin}}F\left(o\left(u\right),Q\right)\\ F\left(o\left(u\right),Q\right)=E_{Q}\left[\ln\;Q\left(s,\pi \right)-\ln\;P\left(o\left(u\right),s,\pi \right)\right] \end{array}$$

The first line of Equation (1) says that actions (*u*) are taken to minimise free energy (*F*) by changing observations (*o*). The second line says that beliefs (*Q*) are also changed to minimise the same free energy functional. The final line defines the free energy in terms of beliefs about states (*s*) and policies (*π*). Policies are simply hypotheses about the alternative state transitions we might actively select (i.e., about alternative trajectories or paths into the future). Policies are sometimes described as sequences of actions, but it is important not to confuse the mental actions in this paper with actions that change the outside world and subsequent outcomes *o*(*u*). The free energy in the final line is formulated as the expected difference between two log probabilities, where the probability distribution indicated by *P* is the generative model; namely, a joint distribution over causes (states and policies) and their consequences (observable outcomes). Typically, forming optimal beliefs about states and policies (i.e., the second line of Equation (1)) is decomposed into two parts:

(2) $$\begin{array}{l} Q\left(s|\pi \right)\leftarrow \min_{Q}E_{Q\left(s|\pi \right)}\left[\ln\;Q\left(s|\pi \right)-\ln\;P\left(o\left(u\right),s|\pi \right)\right]\\ Q\left(\pi \right)\leftarrow \min_{Q}\left(E_{Q\left(\pi \right)}\left[E_{Q\left(s|\pi \right)}\left[\ln\;Q\left(s|\pi \right)-\ln\;P\left(o\left(u\right),s|\pi \right)\right]+\ln\;Q\left(\pi \right)-\ln\;\;P\left(\pi \right)\right]\right) \end{array}$$

The first line can be regarded as perceptual inference, while the second can be read as planning as inference (Botvinick and Toussaint, 2012). Biologically plausible implementations of these equations are usually cast in terms of neuronal dynamics, formulated as gradient flows down free energy gradients. These flows take the following form:

(3) $$\begin{array}{l} {\dot{v}}_{\pi \tau i}^{\left(j\right)}=E_{Q\left(s\backslash s_{\tau }^{\left(j\right)}|\pi \right)}\left[\ln\;P\left(o\left(u\right),s\backslash s_{\tau }^{\left(j\right)},s_{\tau }^{\left(j\right)}=i|\pi \right)\right]-\ln\;Q\left(s_{\tau }^{\left(j\right)}=i|\pi \right)\\ s_{\pi \tau }^{\left(j\right)}=\sigma \left(v_{\pi \tau }^{\left(j\right)}\right)\\ \pi =\sigma \left(-E-G\right)\\ G_{\pi }=o_{\pi \tau }\cdot \left(\ln\;o_{\pi \tau }+C\right)+H\cdot s_{\pi \tau }\\ H_{i}=E_{P\left(o|s=i\right)}\left[-\ln\;P\left(o|s=i\right)\right]\\ s_{\pi \tau i}^{\left(j\right)}=Q\left(s_{\tau }^{\left(j\right)}=i|\pi \right)\\ o_{\pi \tau i}=E_{Q\left(s|\pi \right)}\left[\ln\;P\left(o=i|s\right)\right]\\ \pi_{i}=Q\left(\pi =i\right) \end{array}$$

The final three lines of Equation (3) provide definitions for the elements of the matrices and vectors above. For example, **o**_*πτi*_ is the *i*-th element of the vector **o**_*πτ*_. For full details of these equations, please see (Da Costa et al., 2020; Friston et al., 2017a, b, c) and for didactic treatments see (Bogacz, 2017; Buckley et al., 2017; Parr et al., 2022; Sajid et al., 2021a, b; Smith et al., 2021). However, the key intuitions are as follows. The first equality is a gradient descent on free energy, with a variational posterior parameterised in terms of an unnormalized log probability (**v**). The bracketed superscript indicates a factor of the distribution over hidden states. The second line shows conversion to a normalised probability (**s**) using a softmax or normalised exponential function (*σ*). The third line shows the posterior distribution over policies, with subsequent lines defining the relevant terms. Here, these include the expected free energy for each policy (**G**), and a prior bias (**E**). The **E** and **G** are vectors, whose elements correspond to (the negative log probabilities of) alternative policies. The expected free energy is used to score the implausibility of each policy and does so by penalising *risky* policies whose anticipated outcomes (**o**) deviate from prior preferences (**C**, again, a negative log probability), and whose *ambiguity*—defined as the expected conditional entropy (**H**) of the likelihood (observations given states) distribution—is high. A complementary interpretation of (negative) expected free energy is the combination of expected value (where value is the log preference) and expected information gain, in the sense of Lindley (1956), i.e., a mutual information between causes and consequences given a policy. This can be read as combining the dual aspects of Bayes optimality; in the sense of Bayesian decision theory and experimental design, respectively. The bias term (**E**) is particularly relevant for our purposes as it determines the strength of a (mental) habit. The ‘\’ notation in the equations means ‘excluding’, such that ‘*x* \ *x*_*i*_’ is interpreted as ‘the set of *x* variables excluding the *x*_*i*_ variable.’ In what follows, our focus will be on the parameters **C** and **E**. The **C** parameter can be variously interpreted as a prior preference or motivation, while **E** is interpretable as a habitual bias or cognitive demand.

Although Equations (1), (2), (3)) provide a simple expression of perception and behaviour, they can sometimes seem overwhelming to those unfamiliar with this sort of formalism. One way to develop an intuition for this is to think about it as if it were a serial process—noting that in practice these steps occur in parallel.

1. To begin with, there are several policies we could pursue. To infer the ‘best’ policy, we must calculate the expected free energy—which depends upon our prior beliefs (including our prior preferences)—of each policy.
2. To do this, we need to know the anticipated distributions of both states and observations if a given policy were to be pursued. States may be inferred through belief updating via gradient flows on free energy. Outcomes can then be predicted from the resulting beliefs about states.
3. The expected free energy can be calculated by comparing the anticipated observations with preferred observations and by adding the *risk* of diverging from preferred outcomes divergence to *ambiguity* about which observations can be predicted. Context sensitive beliefs about policies can then be formed, such that the most plausible policies have the lowest expected free energy.
4. Now that we have a distribution over policies, we can then select the actions from the policies that ensure preferred, unambiguous outcomes are realised.
5. New observations then change the free energy landscape, inducing further belief updating. This includes updates in beliefs about policies to assimilate new evidence for which policy should be pursued.

So where do we find a notion of cognitive effort in this belief-updating process? If effort is interpreted as diverging from a cognitive habit, then we can define cognitive effort (**ξ**) as the divergence between context sensitive beliefs about how to act (**G**) and a context insensitive prior belief (**E**), where *Cat* indicates a categorical probability distribution parameterised by a vector of probabilities:

(4) $$\begin{array}{c} \xi ≜\underset{\text{Effort}}{\underbrace{D_{KL}\left[P_{G}\left(\pi \right)\|P_{E}\left(\pi \right)\right]}}=E_{P_{G}}\underset{\text{Context}\;\text{sensitive}}{\underbrace{\left[\ln\;P_{G}\left(\pi \right)\right]}}-E_{P_{G}}\underset{\text{Context}\;\text{insensitive}}{\underbrace{\left[\ln\;P_{E}\left(\pi \right)\right]}}\\ P_{E}\left(\pi \right)=Cat\left(\sigma \left(-E\right)\right)\\ P_{G}\left(\pi \right)=Cat\left(\sigma \left(-G-E\right)\right) \end{array}$$

Why is this useful? The answer is that it tells us which kinds of prior belief are important in determining effort. It can be seen immediately that if context-sensitive priors are the same for all policies (effectively,^2^ **G = 0**), then the precision and demand cancel, and effort attains its smallest value of zero. The **E**-vector encodes prior beliefs about policies, while the **C** and **H**-vectors determine context sensitive beliefs, through their contributions to the expected free energy (**G**, see Equation (3)). This means that effort depends both upon preferences for the fruits of an effortful activity, and the potential information gain from engaging in that activity.

This formulation of cognitive effort suggests that effort is a mixture of context insensitive and context sensitive terms. Cognitive demand depends upon the context insensitive term, which is read here as the prior potential energy (**E**) expected under context sensitive beliefs about policies. In other words, demand is greatest when the expected policy has a high potential or a low habitual probability. The context sensitive term is simply the negative entropy of beliefs about—or confidence in—policies based upon the current context. This means cognitive effort reflects both cognitive demand and uncertainty about the consequences of action. We can consider several scenarios under this construction.

**Fig. 1:**
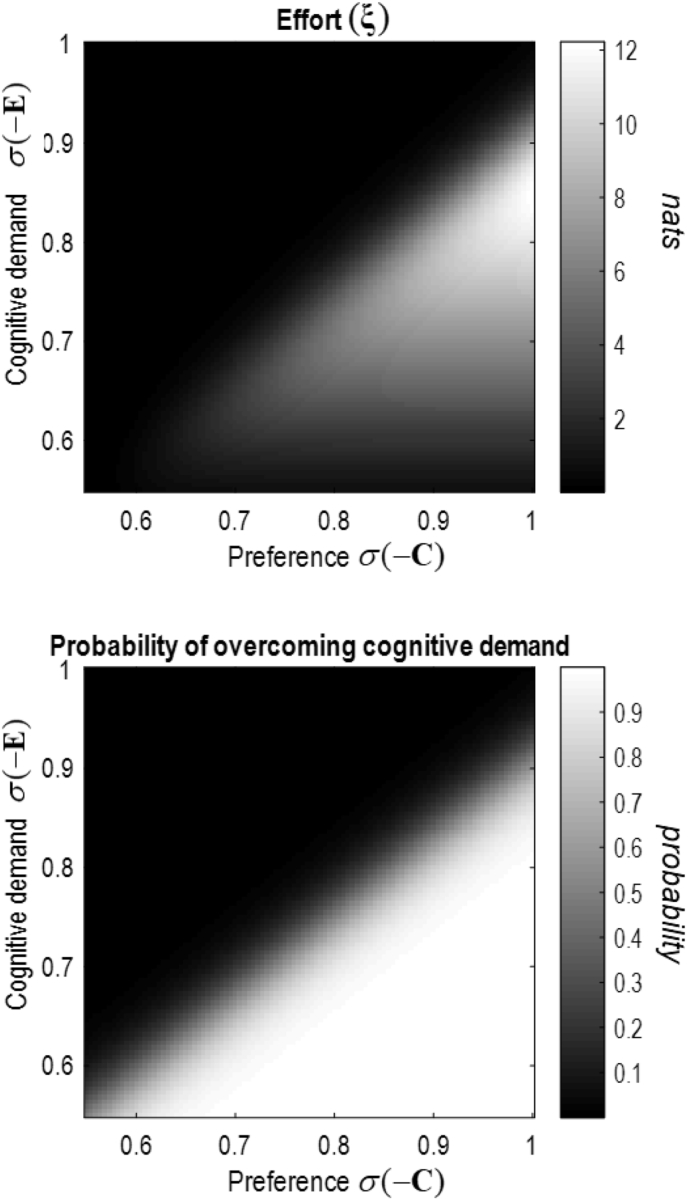
(Cognitive effort). This figure illustrates the central idea of this paper—that cognitive effort may be characterised in terms of the divergence between beliefs about how to act given our mental habits (i.e., the cognitive demand we must overcome) and our beliefs about how we should act under motivational drives (e.g., prior preferences). The plots above are computed by assuming a binary decision in which there is incongruence between the policy favoured by a mental habit and the policy most likely to achieve our prior preferences. The upper plot calculates the cognitive effort for different combinations of prior beliefs, while the lower plot shows the probability of overcoming the mental habit to choose the policy associated with the preferred outcome. In the context of low cognitive demand, the cognitive effort required to ensure the preferred outcome is obtained is minimal. However, a much higher cognitive effort is required with high cognitive demand. When the demand exceeds a certain level, this impairs the deployment of effort, even in the context of strong preferences. Cognitive demand here is the prior probability of the policy that does not fulfil our preferences. The informational units ‘nats’ are the natural-logarithm equivalent of ‘bits’—the informational unit calculated with a base-2 logarithm.

• When the elements of **G** are of similar magnitude to one another, cognitive effort is minimal regardless of **E.** This is because we do not have to update our beliefs much to take account of context. Consequently, the terms in Equation (4) cancel one-another out. One way of thinking about this is that cognitive effort is expended only when we use the expected free energy to contextualise policy selection using goal-relevant information (Dijk and Polani, 2011; Donnarumma et al., 2016). This situation may occur when the reward for a correct response is minimal, in the context of apathy, or when there is no resolvable uncertainty.
• When elements of **G** are large relative to others, the effort deployed depends upon the overlap (or congruency) between **G** and **E.** Maximal effort is required when the two are incongruent, but when elements of **E** are not so large as to preclude any context-sensitive influence over belief updates (see Fig. 1). In other words, cognitive effort must be deployed to overcome a habit that is incongruent with our goals, but a sufficiently strong habit prevents deployment of effort to overcome that habit. Large elements of **G** arise when there is a definitive reward for a correct response or when some actions sate our curiosity.

In short, these parameters give us a space of explanations for measured behaviour in effortful tasks. In what follows, we will manipulate cognitive demand by changing the prior potential (**E**) when it is incongruent with priors based on task demands (**G**). To manipulate both cognitive demand and effort, we will change prior preferences (**C**) that underwrite expected free energy (**G**).

There is nothing special about this definition, and others are plausible. An alternative definition might frame minimal cognitive effort as the point of congruence between habitual and goal-directed priors (i.e., when **E** = **G**), and formulate effort as increasing when the two differ (i.e., when **E** ≠ **G**). The formulation we have adopted is more in line with the idea that effort is a complexity cost, that measures how far we must update our beliefs about how to act when accounting for context sensitivity (i.e., the expected free energy). Normally, a complexity cost is formulated as the divergence from priors to posteriors once observational evidence (a marginal likelihood or free energy) is accounted for. However, for planning, the analogous divergence is from a fixed form prior to a ‘posterior’ distribution that accounts for anticipated observations (via an expected free energy). Practically, Equation (4) simply provides an unambiguous definition of what we mean by effort. Whether this, or another definition, best aligns with subjective experiences of effort is ultimately an empirical question. Note that our formulation is (approximately) consistent with the notion of expected value of control (Shenhav et al., 2013), which offsets the expected value of exerting cognitive control against the cost incurred by exerting that control.

Fig. 1 shows the influence of these parameters on the effort associated with a single binary decision in which the fulfilment of one's preferences is incongruent with the mental habit. The strength of the preferences versus the strength of the habit determines the effort deployed, and the behavioural consequences of this. In what follows, we will unpack this in relation to the Stroop task, in which the ambiguity (**H**) is approximately the same under all policies, meaning we need only concern ourselves with the habitual bias (**E**) and prior preferences (**C**).

**Fig. 2:**
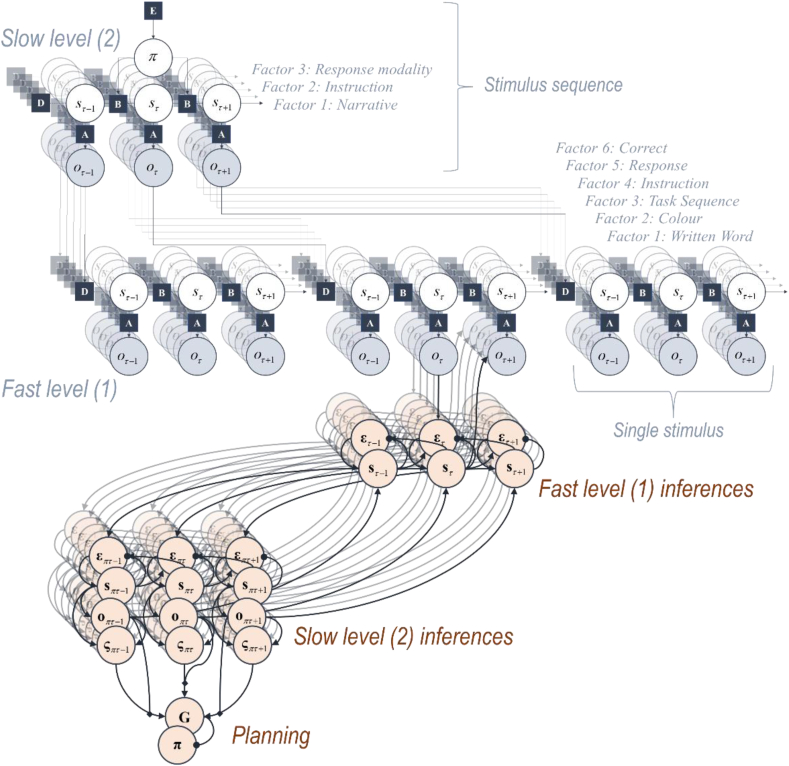
(Deep temporal models). This figure illustrates the architecture of a deep temporal model (blue) and the form of the message passing that implements belief updating under this model (pale orange). The key message to draw from this figure is its symmetry, in the sense that the structure of the message passing (approximately) recapitulates that of the problem. The generative model is displayed as a factor graph (Loeliger, 2004), with squares indicating the factors of various probability distributions. These are labelled **A**-**E** (and **G**) as described in more detail in the main text. Each arrow connects variables (shown in circles) that depend upon one another via that factor. Multiple layers of states and observations are shown, to indicate that there may be many different types of state and more than one outcome modality. In brief, the model we unpack in the main text rests upon a policy (*π*) that determines the transitions among states (*s*). Each state predicts an observation (*o*) which manifests as a sequence of states at a lower, faster, level (much like a word predicts a sequence of letters). The lower-level states themselves generate observations, which are directly accessible to the agent. The message passing is shown such that the observations contribute to prediction errors (**ε**), which depend upon current beliefs about states (**s**) and upon the optimal belief accounting for observations and beliefs about temporally proximal states. These errors are used to update our beliefs, suppressing the error. The expected state in the future is used to predict the next observation (and to generate it when this is controllable by the agent). Beliefs about lower-level states are coupled to those of the slower higher-level states, which are evaluated under each alternative policy. A different sort of prediction error (**ς**), which quantifies the difference between anticipated (**o**) and preferred outcomes, then contributes to the expected free energy (**G**) and posterior expectations about the policy (**π**). Although not shown in the figure, the expectations about the policy are used to average among beliefs about states conditioned upon those policies—effectively, a Bayesian model average. As such, they indirectly influence the next predicted outcome and, therefore, the action taken. In this kind of (variational or marginal) message passing, prediction errors can be read as reporting the free energy gradients that drive belief updating.

Before we discuss the Stroop model in detail, it is worth emphasising the importance of the generative model in determining the form of belief updating and behaviour. For readers interested in technical details, please see (Friston et al., 2017a, b, c). For our purposes, we will note that the belief updating in Equation (3) may be viewed as undoing (i.e., inverting) all the operations performed to generate sensory data—at least, under a model of how they were generated that is entailed by belief updating. This means the sorts of neuronal architecture required to perform this belief updating will mirror the architecture of the generative model (Parr and Friston, 2018). Fig. 2 shows a graphical representation of a (deep temporal) generative model, whose architecture is consistent with the Stroop model we will detail shortly. The neuronal message passing that could invert this model is displayed in pale orange, illustrating the formal (i.e., structural) relationship between a model and its inversion.

**Fig. 3:**
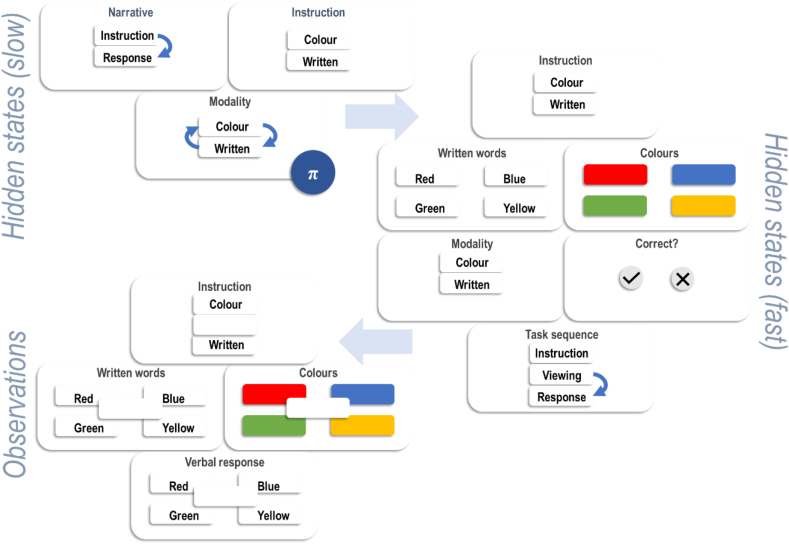
(The Stroop generative model). This figure complements the generic factor graphs and message passing shown in Fig. 2 with the sets of states and observations in our generative model of the Stroop task. This represents the brain's implicit beliefs about how data are generated in the task. This is described in detail in the main text. The light blue arrows show the directional conditional dependencies in the model, while the darker blue arrows indicate allowable transitions. Note that the only policy-dependent state sits at the slow level (as in the factor graph of Fig. 2) and corresponds to the modality chosen to respond with. Some of the slow level states are duplicates of the faster states, enabling inferences about the fast states to be propagated forwards in time (as if held in working memory). The correctness state at the fast level doubles as an observation from the perspective of the higher level and is the only part of the model equipped with preferences. Specifically, there is a preference for being correct. Note that this state has no influence over the outcomes generated, so depends only upon (empirical) prior beliefs.

The upper (blue) part of Fig. 2 depicts a generative model (i.e., a brain's beliefs about the way in which observations are generated). The lettered squares indicate probability distributions, with **E** labelling the context-insensitive habitual prior, **A** labelling the probability of observations given states, and **B** labelling transition probabilities. Each policy is associated with a distinct transition matrix. The **D** vectors give the initial state probabilities. Circles indicate the variables of the generative model—the same variables that appear in Equations (1), (2)). An arrow from one circle to another indicates that the latter is conditionally dependent upon the former. As states and observations are categorical variables, the conditional probability distributions take the form of matrices or tensors. For instance, **A**_*ij*_ = *P* (*o*_*τ*_ = *i* |*s*_*τ*_ = *j*) and **B**_*πτij*_ = *P* (*s*_*τ*+1_ = *i* |*s*_*τ*_ = *j*, *π*). In many models—including that we use for the Stroop task—the states can be factorised into several different state dimensions. In a locomotor model, for instance, we might treat the state as being the (Kronecker) product of location along a North-South axis and an East-West axis. These factors are layered on top of one another in Fig. 2 and are labelled for ease of comparison with Fig. 3. A key feature of this model is its temporal depth, with the observations at the higher (slower) level of the model corresponding to sequences of states at the lower (faster) level. In effect, the higher-level likelihoods (**A**) generate initial states for the lower-level sequences. Equivalently—from the point of view of belief updating—retrospective beliefs about the initial states provide evidence for a particular belief state at the higher (slower) level. The lower (pink) part of Fig. 2 shows a graphical interpretation of the belief-updating detailed in Equation (3) as applied to the generative model. In the next section, we detail the states and observations, and their relationship. Specific choices of **A**-**E**—when substituted into Equation (3)—give us the equations required to simulate belief-updating and ensuing behaviour.

## 3 The Stroop task

In this section, we describe the deep temporal generative model that we used to simulate a simplified version of the Stroop task, in which a participant is asked to either read the word or report the font colour it is written in. The phrase ‘deep temporal’ refers to the hierarchical separation of timescales involved in this model. It means that some things in (the higher hierarchical level of) the model change very slowly, while other things (in its lower hierarchical level) change more quickly. Fig. 2 illustrates this by showing a generative model for which each time step at the higher level is associated with multiple time steps at the level below. In our generative model, the two timescales in question relate to the narrative structure of each trial (the slow scale) and sub-components within each trial (the fast level). At the fast level, we model a trial that begins with a visual stimulus for participants to view, and ends with a response (red, green, blue, or yellow). The slow variables generate faster variables, which themselves generate observable data.

Fig. 3 sets out the overall structure. At the slow (i.e., high) level, there are three hidden state factors. These include a *narrative* state, which changes from an instructional context to a response context; an *instruction* state, which determines whether the task is to read words or to state the font colour; and a response *modality*. The instruction state can be considered equivalent to a ‘task set^3^’. The response modality is the only policy-dependent variable in this generative model and determines the stimulus modality to respond to. This corresponds to what has been referred to as a ‘strategy’ in previous work on the Stroop task (Lovett, 2005). Crucially, this does not influence the external world (i.e., outcomes) directly, so it meets the criteria for a mental action. In this case, the mental action is to determine the response to word stimuli. Either it favours report of the written word or of the font colour. We assume a prior bias towards the former, given this is what we normally do on encountering a written word. Actions that affect the external world, thereby changing outcomes, are specified at the faster level. These actions take the form of vocalisation of a response, detailed below.

The three hidden state factors at the slow level make predictions about the states at the fast level. The latter include the *instruction* and the response *modality*, which are predicted directly by the slow level. In addition, the fast level factors include the *colour* of the font; the *written word*; the *task sequence*; and a state that reports the *correctness* of the chosen mental action. The *task sequence* factor contains three levels: instruction, viewing, and response. If the *narrative* state at the slow level is instruction, the *task sequence* state starts as instruction; if the *narrative* state at the higher level is the response state, the *task sequence* state starts as viewing. The instruction state transitions to itself (i.e., it remains as instruction), while the viewing state transitions to the response state. The *correctness* state reports correct when the *instruction* and response *modality* at the slow level are congruent, and incorrect otherwise. Crucially, this state has no influence over the observations generated, so is inferred from (empirical) prior beliefs only. Prior preferences are set such that correctness is more probable than incorrectness.

The outcomes generated by the lower level include the *instruction*, which is generated only when the *task sequence* hidden state is the instruction. The *instruction* outcome generated depends upon the *instruction* hidden state. During the viewing and response phases of the *task sequence*, a word is generated whose *colour* is consistent with the *colour* hidden state, and whose text (*written word* observation) is given by the *written word* state. During the response phase of the *task sequence*, a verbal response is predicted. This depends upon the *colour* hidden state if the *modality* hidden state is colour, and the *written word* hidden state if the *modality* hidden state is written.

An important feature of this task is that no explicit feedback is given. The preferred ‘correct’ outcome is internalised and plays the role of a first level hidden state. Constructions of this sort are reminiscent of the somatic marker hypothesis (Bechara and Damasio, 2005), which proposes that decision making depends upon preferred interoceptive states. The hypothesis suggests that such states may be simulated by the brain through an ‘as-if’ loop—normally associated with medial temporal or ventral frontal structures. This means there need not be any change in the body, but that decision-making may proceed based upon the sensory data we would have received as if we were receiving sensory feedback about these decisions.

The temporal structure of the model is important in that it allows for inferences about slowly-changing variables (including the task *instruction*) to be maintained over the course of sequences of fast-changing variables (including the *colours* and *written words*). This facilitates a form of working memory, sometimes referred to as a ‘semi-Markovian’ model (Marković et al., 2021).

**Fig. 4:**
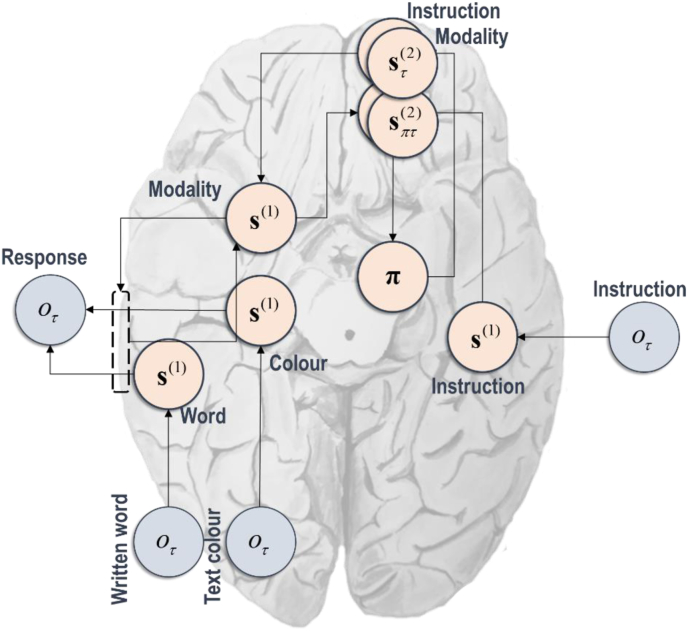
(Computational anatomy). This figure is intended to convey some intuition for the architecture of the inferential message passing required to perform belief updating under the model of Fig. 3, and to suggest how it might manifest in neural circuitry. For simplicity many of the connections, and some of the intermediate nodes, from the lower part of Fig. 2 have been omitted. In addition, the lateralisation should not be taken seriously—some nodes have been placed on one side of the graphic simply to avoid visual clutter. In brief, the instruction outcome informs first level beliefs about the instruction via the auditory cortices in the temporal lobes. These beliefs are propagated to the second level, where slowly changing beliefs about the instruction and the intended response modality are held. Slowly changing neural activity, of the sort we would anticipate being associated with these beliefs, is often associated with the prefrontal cortices (Fuster, 1973). Beliefs about sequences and narratives (omitted from the figure) are sometimes associated with the hippocampi (Foster and Wilson, 2007; Frölich et al., 2021; Huerta and Rabinovich, 2004; Pezzulo, Kemere and van der Meer, 2017), which share reciprocal connections with the prefrontal cortices. At the second level, beliefs about the instruction are used to formulate beliefs about the intended response modality under each alternative policy, and the (first level) consequences of these alternative choices, perhaps involving the anterior cingulate cortex (Scherbaum et al., 2012; Shenhav et al., 2013, 2017). These consequences are used to formulate beliefs about the most appropriate policy (i.e., the policy that ensures the preferred ‘correct’ state at the first level), ultimately favouring the policy in which the instruction and modality match. The policy then weights the conditional beliefs about the modality, resulting in a Bayesian model average. The relationship between conditional beliefs, policies, and Bayesian model averages, has previously been noted to have a similar architecture to cortico-basal ganglia circuitry (Friston et al., 2017a, b, c). At this point, beliefs about the policy may also be influenced by other prior biases—here that the most informative modality from a visual stimulus to a verbal response is the writing itself, and not the colour of the text. In other words, we have a prior bias towards reading written words. Beliefs about the modality at the second level are then propagated to the first level as prior beliefs about the modality that determines the response. Beliefs about the colour and the word are formulated based upon visual data through colour (V4 (Pasupathy et al., 2020)) and word (visual word-form (McCandliss et al., 2003)) regions of the extrastriate cortices. Their ultimate influence on the verbal response depends upon the first level modality beliefs. For example, if the modality is believed to be the written word, then the written word hidden state is assumed to precisely predict the response outcome.

Fig. 4 provides a heuristic expression of the computational anatomy involved in solving this task. Although primarily to aid intuition as to the mechanics of the active inference scheme employed, it also serves to illustrate how the message-passing architectures illustrated in Fig. 2 can be used to motivate neuroanatomical hypotheses. This is important from a computational neuropsychology perspective, as it lets us relate anatomical lesions to aberrant prior beliefs, we might anticipate following damage to this part of the network. For instance, if we associate posterior beliefs about the mental policy (to select a response *modality*) with the output of the basal ganglia, we might anticipate damage to parts of this subcortical circuit would change the prior bias towards reading, as opposed to naming the colour of, text. In contrast, when we associate beliefs about the slowly changing variables with the prefrontal cortices, we might anticipate that our ability to predict the *correct* state would deteriorate with damage to these cortices (Parr et al., 2019), giving the appearance of a reduced preference for being correct. This anatomy may or may not be a good hypothesis as to the computational neuropsychology of cognitive effort. However, if we were to subscribe to this hypothesis, it predicts that we can associate the determinants of cognitive effort with the basal ganglia (**E**) and the prefrontal cortices (**C**).

## 4 Simulated behaviour and electrophysiology

For the simulations that follow, the prior bias is set such that 85% of the time, our synthetic brain expects to read the word presented to it, and 15% of the time, it expects to name the colour. The prior preferences are set such that, prior to normalisation, being correct has a log probability of 1 and being incorrect has a log probability of −1. This means there is a prior belief in place that the mental policy will be selected such that being correct is *e*^2^ (≈7.4) times more probable than being incorrect. This can be read as the degree of preference for being correct, which is weighted against the strength of habitual policies in predicting the likely outcomes. Overt actions are selected based upon the predicted distribution over outcomes. In other words, outcomes are realised by overt actions based upon the subject's predictions:

(5) $$\begin{array}{l} o_{\tau +1}∼Cat\left(u_{\tau }\right)\\ u_{\tau }=\sigma \left(\lambda \;\ln\left(A\right)s_{\tau +1}\right)\\ s_{\tau +1}=\sum_{\pi }\pi_{\pi }s_{\pi ,\tau +1} \end{array}$$

Note that the **s** variable is a function (Bayesian model average) of beliefs about policies. This means that policies are inferred, as opposed to ‘selected,’ but do influence the actions that go on to be selected. This explicitly disambiguates the processes of planning and acting. The *λ*-parameter is an inverse temperature parameter that determines the degree of stochasticity in action selection. Equation (5) is a relaxation of the first line of Equation (1)—which is recovered for very large *λ*. If *λ* is much smaller, more randomness is introduced. We use *λ* = ¼ to account for the fact that this model is not intended as a model of everything that is going on in the brain, and there may be other computations going on that could influence action selection. In place of simply selecting the most probable action, the overt action involves sampling the next observation from a distribution given by a softmax function of the free energy for the next time step. The only part of the free energy that depends directly on observation is the expected log likelihood (or accuracy), expressed here in linear-algebraic terms. This expected observation in turn depends upon the covert mental action, which underwrites beliefs about the future.

**Fig. 5:**
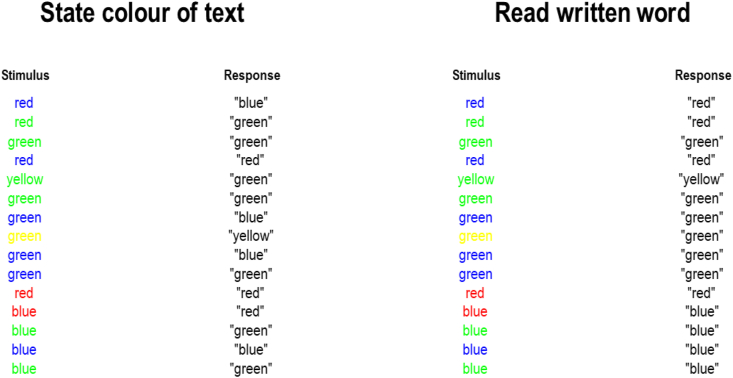
(Simulated behaviour). The rows in this figure show the sequence of stimuli presented, and the response given to each stimulus during simulation of an artificial agent with the generative model outlined above. In the left column, when the task is to report the font colour, the responses are nearly all correct, with two exceptions. When incorrect, the responses are consistent with what would have been correct in the reading condition. Interestingly, the incorrect responses follow from correct responses when the word and the colour are congruent—i.e., the previous response would have been consistent with either modality. In the written word condition (shown in the right panel), all responses are correct. For the word-reading condition **ξ** ≈ 5.5 nats for each decision, while for the colour-naming condition **ξ** ≈ 7.0 nats for each decision. Interestingly, the effort deployed (**ξ**) does not vary with congruency, offering a dissociation between effort and performance which, as we will see later, is enhanced in the congruent condition.

Fig. 5 shows the behaviour generated by inverting the generative model under the two alternative instructions (i.e., report the colour of the text or read the written word). Practically, this means setting the instruction hidden state of the data-generating process. The prior belief held by our synthetic participant is not changed between these conditions. Note that responses are 100% correct when the task is to read the word, but there is an error following the fourth stimulus when the task is to report the font colour. For this stimulus, the word ‘red’ is read out, while the correct response would have been ‘blue’, which is the font colour. There is also an error for the tenth stimulus. It is significant here that both of these error responses are the written words, so are not simply random errors. This is consistent with the ‘interference’ phenomenon originally noted during this task (Stroop, 1935). From our perspective, it represents insufficient deployment of cognitive effort to overcome cognitive demands (see Fig. 1). Mathematically, this means that **E** dominates Equation (3), having more influence relative to **G**. In other words, the mental habit of reading is stronger than the context sensitive (i.e., instructional set) motivation to perform the task correctly. The perfect performance in the reading condition is consistent with the lower cognitive demand of this task, which is consistent with what we expect to do 85% of the time even without preferences and instructions.

An interesting observation from Fig. 5 is that the incorrect responses do not seem to occur at random but following correct responses to congruent stimuli. Intuitively, this is because the correct response for congruent stimuli provides evidence for both colour-naming and word-reading conditions. In contrast, the correct response for incongruent stimuli provides definitive evidence for one instruction over the other. When the evidence is ambiguous for one stimulus, the relative uncertainty about the instruction is then propagated to the next stimulus, making it more likely that an error will be made. This emergent property of our simulations—that responses may depend upon previous elements of the stimulus sequence—coheres with an established phenomenon referred to as the congruency sequence effect (Botvinick et al., 2001; Schmidt et al., 2015; Shenhav et al., 2013). It also implies that the pattern of responses may be significant in drawing inferences based upon behavioural data—not just the overall accuracy statistics that are often used to characterise behaviour in Stroop tasks.

As detailed in the figure legend, we can compute the effort associated with performance based upon Equation (4). As expected, the colour-naming condition requires greater deployment of effort than the word-reading condition. Interestingly, there is no variation in effort with congruency. This initially seems at odds with observations that incongruent trials are subjectively experienced as more aversive (Dignath and Eder, 2015; Dreisbach and Fischer, 2012). However, while related, it is important to distinguish between something being aversive and effortful. Aversion would be more relevant in this task if choices influence whether they saw more congruent or incongruent stimuli. Given the greater chance of fulfilling one's preferences—when stimuli are congruent—we would expect a larger expected free energy associated with choices leading to the incongruent versus congruent condition, resulting in (the behavioural signs of) an aversion to incongruent stimuli. It may be that it is difficult to disambiguate between the subjective experiences of effort and aversion, and both may be at play in this task. With clear definitions of each quantity, there is scope to disambiguate between these through estimation of the associated parameters in a generative model. Based upon the simulations here, subjective experiences of aversion to incongruent stimuli would not be consistent with variations in effort as defined above.

**Fig. 6:**
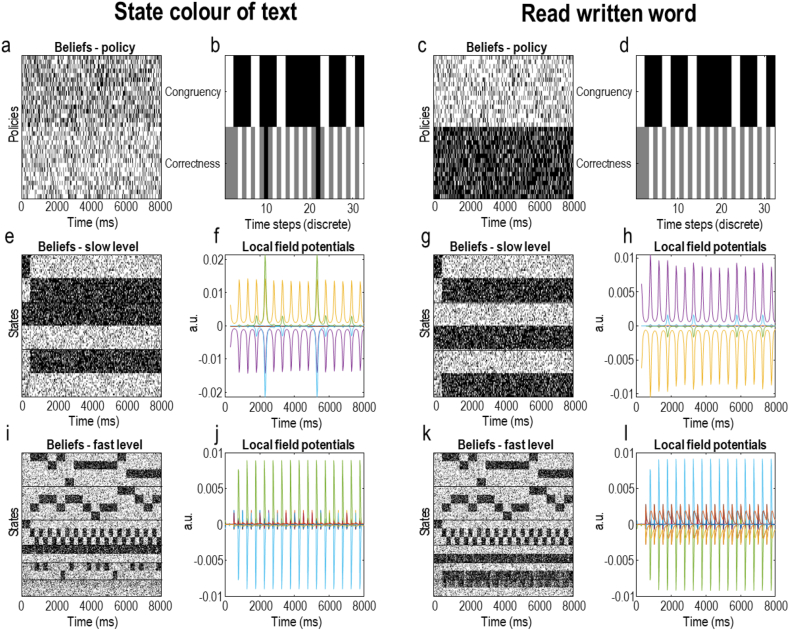
(Synthetic neurophysiology). Exploiting the duality between inferential dynamics and physiology (sometimes characterised as a Markovian monism (Friston et al., 2020)), we can examine the belief-updating process as it might manifest in neuronal activity. These plots show the same sequences as in Fig. 4 but offer an insight into the mechanisms that generated the behaviour seen in the previous figure. The 6 plots on the left (a, b, e, f, i, j) relate to the colour-naming condition and the 6 plots on the right relate to the word-reading condition (c, d, g, h). Panels b and d depict the congruency of the stimuli and whether or not a correct response was given at each time-step (each trial comprises 2 time-steps—one for viewing and one for responding to the stimulus). White indicates congruency or correctness, black indicates incongruency or incorrectness, and grey indicates time-steps at which no response was given. The plots depicting beliefs (a, c, e, g, i, k) are formulated as raster plots. Each row represents a neuron, with spikes shown in black, and the absence of a spike shown in white—each neuron is replicated 16 times as if we had run the experiment identically 16 times and measured the response of that neuron. These spikes are generated by sampling from the posterior probabilities inferred through free energy minimisation, under the assumption that the average population activity of subsets of neurons encodes these probabilistic beliefs. The black horizontal lines in the belief plots separate each hidden state factor. The factors in the fast level are ordered as follows: *written word*, *colour*, *task sequence*, *instruction*, *response*, *correct.* In the slow level, the order is: *narrative*, *instruction*, *response.* Some key observations are as follows. First, the hierarchical model ensures beliefs about the first level states evolve much faster than those associated with the second level states or policies. Second, note the first level neural population that shows an alternating firing rate pattern, representing the alternation between viewing and responding to a stimulus. Third, the distinction between the two policies under the colour-naming condition (a) is less definitive than in the word-naming condition (c). This is because the colour-naming condition involves two conflicting sources of information: a belief that correct responses will be given if the instruction is followed and a prior belief that word-reading is a more common policy. In contrast, when the instruction is consistent with prior beliefs, greater confidence can be obtained. The local field potential plots depict the (filtered) rates of change of posterior beliefs.

An advantage of having a process theory associated with the belief-updating—that generated the behavioural responses in Fig. 5—is that we can examine these belief updates as we might examine neuronal responses. Fig. 6 shows the electrophysiological correlates of these inferential dynamics and offers some hints as to the mechanisms that underwrite the behaviour in Fig. 5. The belief plots (Panels 6a, c, e, g, i, and k) show samples from either **π** (for beliefs about policies) or **s** (for beliefs about states) as if we were measuring spikes from single neurons from a population of neurons encoding these variables with their average firing rates. The associated local field potentials (Panels 6f, h, j, and l) are generated from the rates of change of the firing rates, with the highest frequencies suppressed to eliminate the effects of the artificial discretisation in time. The two key things to draw from Fig. 6 are (*i*) the much faster change in belief states at the first (faster) level of the model (lower row of plots) compared to the second (slower) level (middle row of plots), (*ii*) the differences in the amplitudes of the local field potentials associated with congruent and incongruent stimuli (and correct and incorrect responses), and (*iii*) the prior bias in policy selection means that beliefs about policies are more precise (i.e., shown as a difference in shading between rows) in the word-reading condition compared to the font-colour condition. The most obvious place to see the second of these observations is the local field potential for the neuron shown in green in Panel 6f. Note the small increase in amplitude whenever the stimuli are congruent (as indicated in Panel 6 b), and the much larger increase when a response is incorrect—consistent with the ‘error-related negativity’ (Yeung et al., 2004), an increased amplitude of evoked response with erroneous as opposed to correct responses.

**Fig. 7:**
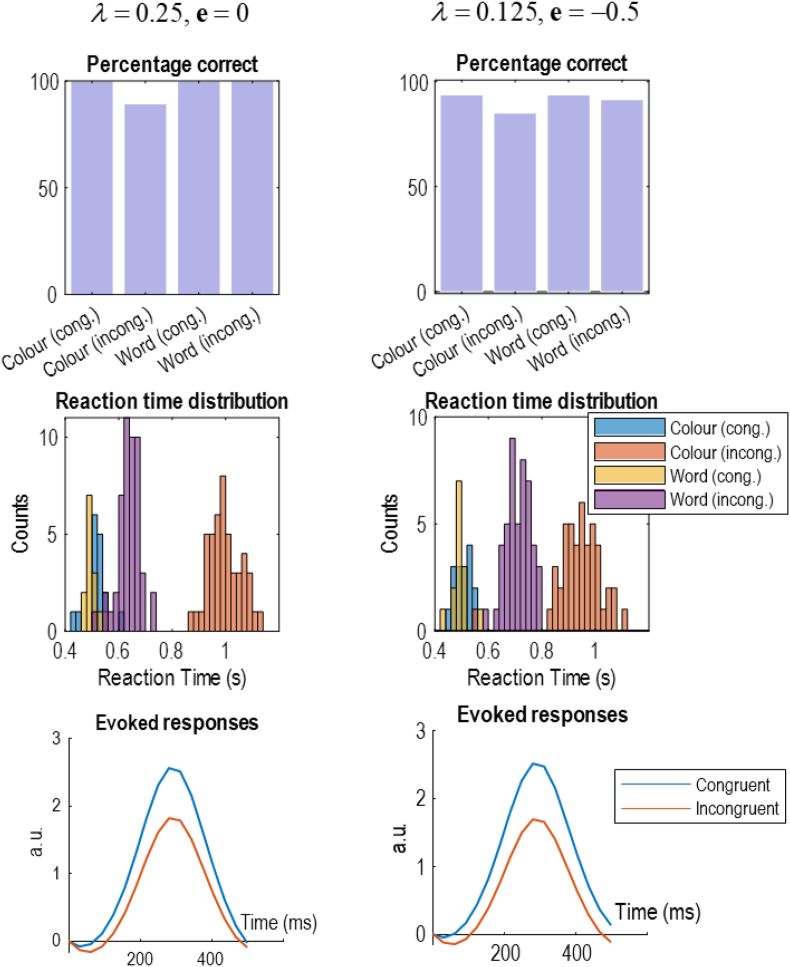
(Condition specific effects). This figure offers a construct validation of this model against data measured from experiments using the Stroop task. The two columns reproduce the same measurements under different parameter settings. Here, we simply illustrate that different behavioural and electrophysiological patterns can be reproduced with different parameter values. The changes from the left to the right column are a reduction in the strength of the habitual bias and reduced precision in response generation, which together increase response variability. The parameters used here were chosen to emphasise the qualitative differences between conditions characteristic of the Stroop task. The upper plots show the proportion of errors in different conditions. In the left plot, no errors were observed in the congruent conditions, or in the word-reading condition. However, performance was worse during the font-colour condition when the stimuli were incongruent. A more nuanced pattern is shown in the right plot, with worse performance in both incongruent conditions relative to congruent and worse performance in the incongruent colour-naming condition specifically. The middle plots show the reaction time distributions for each condition. Reaction times were modelled as detailed in the main text. This shows similar distributions in the congruent conditions, but longer reaction times in the incongruent conditions, with font-colour naming requiring a longer reaction time than word-reading. The lower plots show the evoked responses in the font-colour condition averaged over trials within congruent and incongruent stimulus presentations. The evoked responses are simply the local field potentials as shown in Fig. 6 taken from the second level neuronal populations representing the response modality. Electrophysiologically, the Stroop effect is often characterised by greater amplitude of congruent relative to incongruent waveforms (Badzakova-Trajkov et al., 2009), consistent with the simulated waveforms in this plot.

Fig. 7 shows the types of descriptive statistics that are often presented for empirical data, for the simulations described above. In the left column of plots in Fig. 7, the simulations in Fig. 5, Fig. 6 have been extended to 64 stimulus presentations (including the initial instruction). We have plotted the percentage correct, the reaction time distribution, and average evoked responses. From the data in Fig. 7, it is clear that performance of the task is 100% when colour and word stimuli are congruent, and also in the incongruent condition when the task is to read the written word. However, in the incongruent condition when the task is to name the font colour, there are some errors. Therefore, this simulation reproduces the Stroop effect: When the task is to name the font colour, there is a difference in accuracy between congruent and incongruent trials (that is not present when the task is to read the word).

Depending upon the precise trial design, we might not expect 100% response rates in the incongruent word-reading condition (or even in the congruent conditions). For example, if participants are asked to respond very quickly, we might expect a small number of errors in all conditions. This increased error rate can be simulated by decreasing the *λ*-parameter from Equation (5). For example, when *λ* and **e** are relatively small (e.g., 1/8 and −1/2, respectively)—corresponding to an increasingly difficult task with only a slight word-reading bias—we observe a small number of errors in all conditions. However, the pattern of errors is non-unform; with many errors in the incongruent colour-naming condition, some in the incongruent word-reading condition, and very few in the two congruent conditions. This is shown in the right column of plots in Fig. 7.

The same interaction between instruction and congruency is displayed for the reaction time distributions. Before unpacking these, it is worth briefly outlining the way in which reaction times are generated, as this deviates from previous active inference process theories. Previously, reaction times have been computed simply by timing how long it takes a computer to simulate the neuronal message passing. While this has been sufficient to reproduce some simple behavioural phenomena, it does make reproducibility (and model fitting) challenging, as different computers at different times might take different lengths of time to perform the same computation. In this paper, reaction times are based upon confidence, as is common in modelling reaction times (Feltgen and Daunizeau, 2021; Ratcliff and McKoon, 2008). The basic idea is that confidence, or precision, manifests biologically in synaptic time constants (Feldman and Friston, 2010). Increased precision leads to faster neuronal computation, and therefore faster response times. Specifically, the reaction time is taken to be a function of the entropy of the predicted verbal outcome at the next time step. Entropy and precision are inversely related, so greater entropy implies longer reaction times. The reaction time distributions in Fig. 6 are constructed by sampling from the following process:

(6) $$\begin{array}{l} r_{\tau }=\frac{1}{2}\exp\left(n-u_{\tau +1}\cdot \ln\;u_{\tau +1}\right)\\ n∼N\left(0,\frac{1}{256}\right) \end{array}$$

For those familiar with drift-diffusion modelling of reaction times (Ratcliff and McKoon, 2008), the negentropy in Equation 6 can be thought of as the (log) drift rate that determines the decision time, with the **n** variable accounting for the influence of diffusion. The log normal distribution ensures no negative reaction times. Note that the range of reaction times resulting from Equation 6 are approximately the same ranges as measured empirically (Coderre et al., 2011). The qualitative relationships between reaction times to incongruent and congruent stimulus presentations are also consistent, with longer reaction times when the stimuli are incongruent. Note the longer reaction times associated with incongruent colour-naming compared to incongruent word-reading, consistent with our prior bias towards reading words.

The lower plot in Fig. 7 is generated by averaging the local field potentials during the font-colour task in the congruent and incongruent conditions. The greater amplitude response in the congruent conditions reproduces qualitative empirical findings (Badzakova-Trajkov et al., 2009; Coderre et al., 2011). The neuronal populations responsible for this evoked potential are those associated with the response modality at the second level—i.e., the controllable state that represents the cognitive policy. This reflects the fact that both response modalities are afforded evidence by the congruent stimuli, promoting belief updating when only one of the two modalities was previously thought plausible. The behavioural correlate of this is the higher tendency to make an error when an incongruent stimulus presentation follows a congruent presentation, remarked upon above in relation to Fig. 5.

## 5 From priors to behaviour

We next turn to the question of how variation in the cognitive demand or cognitive effort between subjects, or between the same subject under different experimental (e.g., pharmacological) manipulations, manifests in behaviour. This analysis is in the spirit of Musslick et al. (2019) who used a similar approach to associate model parameters with behavioural measures. To do this, we simulated task performance in the font-colour condition for 25 subjects who have different prior beliefs (i.e., with different **C** and **E** parameter combinations). We formulated these differences using log scaled versions^4^ of the parameters outlined above, where **e** can be conceptualised as cognitive demand and **c** can be conceptualised as the motivation to overcome this demand (i.e., the preference for being correct):

(7) $$\begin{array}{l} C\propto \exp\left(c\right)\times {\left[\begin{array}{cc} -1 & 1 \end{array}\right]}^{T}\\ E\propto \exp\left(e\right)\times {\left[\begin{array}{cc} -0.85 & 0.85 \end{array}\right]}^{T} \end{array}$$

**Fig. 8:**
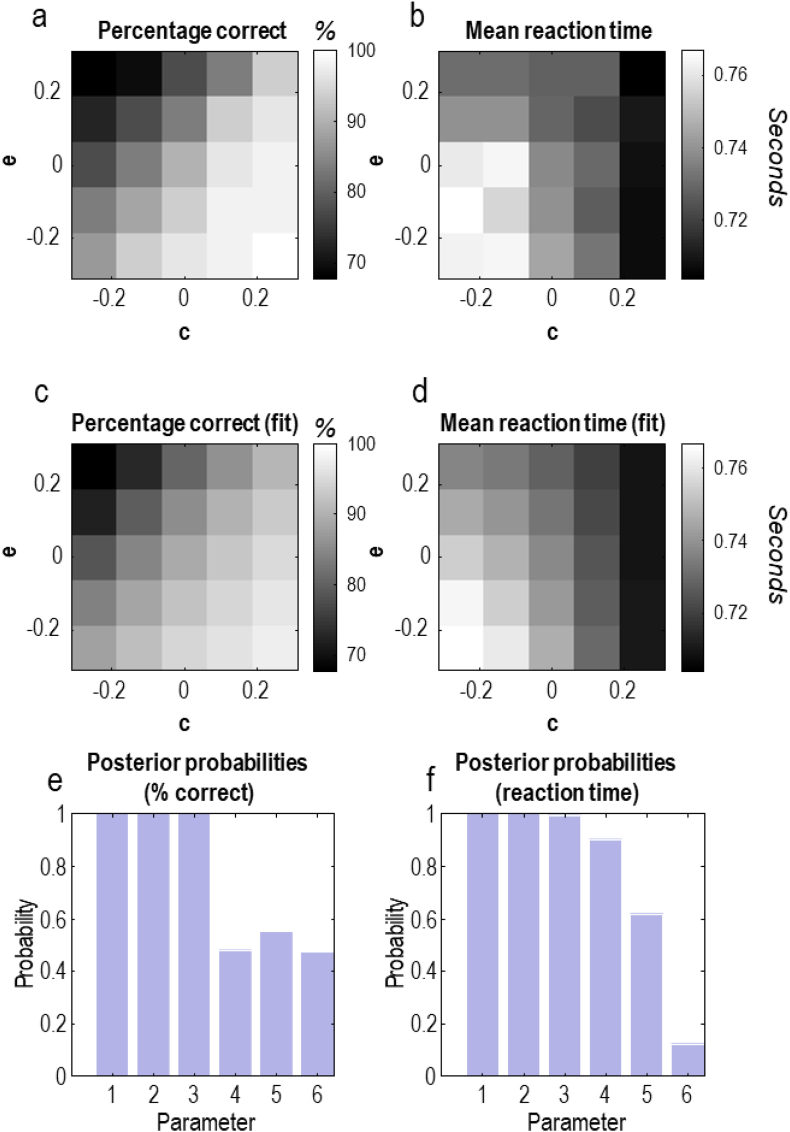
(From priors to behaviour). This figure illustrates the effect on average reaction times and error rates in each condition when prior beliefs are varied to either make the task more demanding (**e**) and to encourage a greater deployment of cognitive effort (**c**). The upper plots show the results of simulating behaviour under each of these priors. The middle plots illustrate the pattern when we fit the models of Equation (8) (i.e., the *β-*coefficients) to these simulated data. The lower plots show the results of an application of Bayesian model reduction to these models to determine the relevance of the *β*-parameters in explaining the simulated data. Specifically, we compare the evidence for all models with and without a given parameter in play and use this to compute the posterior probability of models with the parameter. The interpretation of these results is that there is good evidence (posterior probability ≈ 1) to suggest the first three parameters (i.e., a constant term, the influence of **c**, and the influence of **e**) are needed to explain the proportion correct data. However, the data are uninformative about (i.e., provide no evidence for or against) the importance of parameters representing the second order terms. Reaction times are better explained by the constant, linear, and interaction (**c×e**) terms, with evidence against a non-zero **e**^2^.

The upper plots in Fig. 8 report the percentage correct responses (Panel 8a) and the average reaction times under different combinations of these priors (Panel 8 b). These plots show that percentage correct becomes worse as cognitive demand increases but becomes better as the motivation to deploy cognitive effort increases—as we would intuitively expect. There is a slightly more complex relationship between the two parameters for reaction times. Nevertheless, the fastest reaction times are found in the context of low demand and high effort, as we would expect. Note that each cell in Panels 8a and b corresponds to an individual simulated subject who has different values for **c** and **e**; here, we express cognitive demand (**e**) not as a fixed property of the task itself, but how demanding a given subject finds the task, which is not necessarily the same for all simulated subjects. The relevance of this is that we present all participants with exactly the same task sequence, implying any difference in cognitive demand relates to the subject, not the task.

To gain a greater understanding of these relationships, we tested a series of hypotheses about the contributions of these prior parameters to (simulated) behaviour. First, we used variational Laplace (Friston et al., 2007) to estimate the coefficients of the following models:

(8) $$\begin{array}{l} P\left(y_{c}|\beta_{c},\lambda_{c},c,e\right)=N{(\sigma \left({\left[X\left(c,e\right)\beta_{c},1\right]}^{T}\right)}_{1},\exp\left(-\lambda_{c}\right))\\ P\left(\beta_{c}\right)=N\left(0,I\right)\\ P\left(\lambda_{c}\right)=N\left(4,1\right)\\ P\left(y_{r}|\beta_{r},\lambda_{r},c,e\right)=N\left(X\left(c,e\right)\beta_{r},\exp\left(-\lambda_{r}\right)\right)\\ P\left(\beta_{r}\right)=N\left(0,I\right)\\ P\left(\lambda_{r}\right)=N\left(4,1\right)\\ X\left(c,e\right)=\left[\begin{array}{cc} \begin{array}{cc} 1 & c \end{array} & \begin{array}{cccc} e & c\times e & c^{2} & e^{2} \end{array} \end{array}\right] \end{array}$$

The data *y*_*c*_ are the proportion correct and *y*_*r*_ are the log mean reaction times. The softmax function ensures the expected proportion correct lies between 0 and 1, while the use of the logarithm for the reaction times ensures the expected reaction time is positive. The models above can be thought of simply as linear models of the kind we might use in a regression analysis, but with non-linearities applied to ensure the model outputs conform to the allowed ranges for the available data. The *β*-coefficients in the above can be estimated for simulated data, as shown in Fig. 8.

Estimating the values of the *β*-coefficients (and the *λ*-parameters) allows us to ask which of these coefficients are important in explaining the synthetic data. Specifically, we can ask whether the coefficients have values that are non-zero. If not, this would effectively mean removing these parameters from the equation (i.e., ‘pruning’ them away). To do this, we specify priors over combinations of *β*-coefficients as precisely zero and examine whether the marginal likelihood increases or decreases—thereby allowing us to estimate whether the parameter is useful for explaining the data (i.e., proportion correct or reaction times). Bayesian model reduction (Friston et al., 2018; Friston et al., 2016) is a statistical technique used to perform these comparisons quickly and efficiently.

The middle plots of Fig. 8 show the patterns of behaviour predicted under the models of Equation (8) using the posterior modes of the *β*-coefficients. The lower plots show the (posterior) probabilities that each *β*-coefficient is non-zero. These are computed for each parameter by summing the posterior probabilities for various models in which that parameter was allowed to vary from zero. Here, we find evidence (with a posterior probability approaching one) for an effect of **c** and **e** (and an additional constant term) on the proportion correct, but no convincing evidence for higher order interactions (posterior probability ≈ 0.5). The reaction times are best explained when effects of the interaction **c×e** (posterior probability ≈ 0.9) and of the quadratic term **c**^2^ (posterior probability ≈ 0.6) are included in the model. All other effects are associated with a posterior probability of <0.5, implying evidence against non-zero values for the associated coefficients. The **c×e** interaction suggests that reaction times are not simply an additive combination of **c** and **e**; instead, the fastest responses are made when greater motivation is deployed in the context of a high cognitive demand.

A final point to note about the plots in Fig. 8 concerns the relationship between reaction time and accuracy with variations in each parameter. As seen most clearly in panels 8c and 8 d for small **c**, those values of **e** for which reaction times are longer are also those for which accuracy is greater. For larger values of **c**, this relationship is abolished. The implication is that, when preferences are relatively weak, variation in **e** leads to a speed-accuracy trade-off of the sort frequently encountered in tasks requiring cognitive control (Bogacz et al., 2010; Chittka et al., 2009; Drugowitsch et al., 2012; Heitz, 2014; Henmon, 1911). As preferences for being correct become stronger, the speed-accuracy trade-off is first attenuated, and then the relationship between the two disappears. This is interesting in the context of empirical work (Manohar et al., 2015) demonstrating the disruption of the trade-off when the incentive for correct responses (i.e., the strength of preferences) is increased. As **c** increases from left-to-right in 8c and 8 d, the same pattern is seen. There is a straightforward explanation for this phenomenon under the model presented here. As reaction times are assumed to be a function of confidence in one's next action, we see that confidence will be high when there is a strong prior habit (i.e., when **e** is large) provided there are not strong preferences that contradict this (i.e., when **c** is small). In the extreme case, this means we can confidently and quickly perform our habitual (word-reading) action every time. However, this will clearly result in lower accuracy. As **e** becomes smaller, confidence in our next action declines, but accuracy will increase as we cease to be biased by our cognitive habits. The implication is that variance in **e** at small **c** will necessarily lead to a speed-accuracy trade-off. Such results have implications for a wide range of studies examining variations in movement vigour with changes in the value attained following these movements (Reppert et al., 2015; Summerside et al., 2018; Yoon et al., 2018).

## 6 Computational phenotyping

Having demonstrated that behavioural measures can be predicted based upon the prior beliefs of the behaving subject, we now turn to the question of whether we can infer these prior beliefs from behaviour. This is not a straightforward problem, as is evident in the plots of Fig. 8, in which different combinations of parameters lead to the same behavioural measures. This pattern of results is intuitively sensible, as we might expect similar behaviour in a cognitively demanding task in which a great deal of cognitive effort is deployed and in a less cognitively demanding task in which less cognitive effort deployed. The implication is that there is a many-to-one mapping from prior parameters to behaviour, and that the problem of inferring priors from behaviour is an example of an inverse problem.

Such problems are common. Examples include the problem of inferring the voxels responsible for patterns of measured electromagnetic activity on the scalp in electroencephalography or magnetoencephalography research, or the 3-dimensional geometry of an object based upon the photoreceptor activity on a retinal sheet. Almost invariably, these problems call upon Bayesian inference for their solutions (Baillet and Garnero, 1997; Calvetti and Somersalo, 2018; Watzenig, 2007). The reason for this is that the prior plausibility of each parameter of the model (here, the **c** and **e** parameters), required for Bayesian inference, enforces a unique solution. This is not always the ‘correct’ solution—in the sense of recovering the parameters used to generate the data—but is the best explanation for the data available. The notion of ‘best’ here accounts for the fact that the data generated by any given model could also be generated by a more complicated model. However, Occam's razor favours the explanation that—simultaneously—is the simplest and most accurate account of the data.

To infer the **c** and **e** parameters, we specified prior beliefs that the two parameters were distributed according to normal distributions, each with zero mean and prior variances of 1/126. The small prior variances are based on the results in Fig. 8, which show that the model is highly sensitive to variations in the parameters. Small increases in **c** or decreases in **e** from their prior values lead to 100% accuracy. If the parameters are outside of the range that accuracy and reaction times vary, then model inversion will not estimate the parameters with high certainty—because the data are ambiguous. For example, this would be the case for hypothetical populations who perform the Stroop task perfectly. We can assume that this task is only useful for inferring parameters related to effort when at least some errors are made. The log likelihood function for this model is obtained by forcing the active inference scheme to select the same actions as were chosen with the (synthetic) behavioural data and presenting it with the same sensory data. In many analyses of the Stroop task, choice data are usually summarised in terms of the accuracy with which the task is performed. However, our modelling approach makes use of not only the overall accuracy, but of the sequence of choices (i.e., verbal responses) made. This allows for sequential effects to inform the fitting of the model to data. The log likelihood is influenced by both the responses that were made and the speed of the responses, given as:

(9) $$L\left(c,e,o,r\right)=\underset{\text{Choices}}{\underbrace{\sum_{\tau }\;\ln\left(o_{\tau }\cdot u_{\tau -1}\left(c,e,o_{t\leq \tau -1}\right)\right)}}-\underset{Re\text{action}\;\text{times}}{\underbrace{\sum_{\tau }\frac{1}{256}{\left(\ln\;r_{\tau }+u_{\tau }\left(c,e,o_{t\leq \tau }\right)\cdot \ln\;u_{\tau }\left(c,e,o_{t\leq \tau }\right)+\ln\;2\right)}^{2}}}$$

**Fig. 9:**
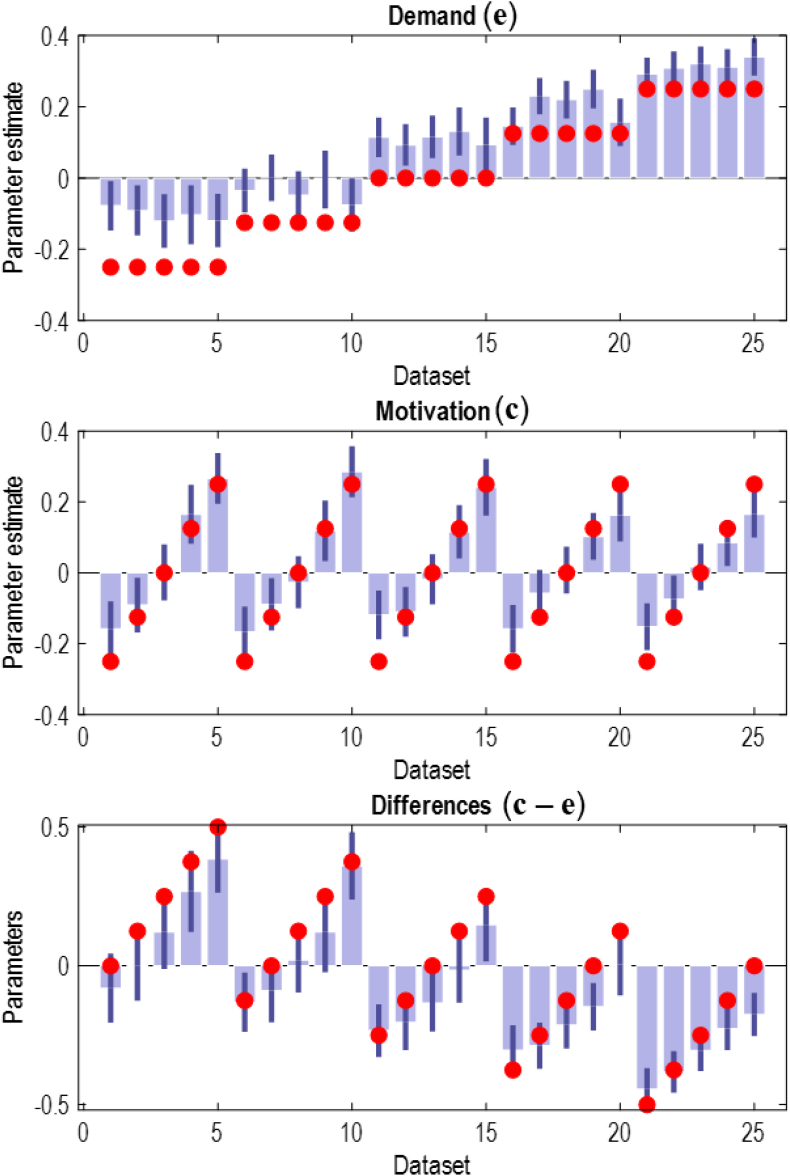
(Model fits). The plots in this figure illustrate the results of fitting the active inference scheme, using the generative model of Fig. 3. The red markers indicate the parameters used to generate a synthetic behavioural sequence (and set of reaction times), with the blue bars indicating the expectation and 90% credible intervals associated with the estimates. The **e** parameter is shown in the upper plot (labelled ‘demand’). The **c** parameter is shown in the middle (motivation) plot. The final plot shows the difference between **c** and **e**, accounting for the posterior covariances. Note that, while the estimation of **e** is less accurate at lower values, the differences between the two is better estimated. This reflects the fact that, while there is no unique parameter combination that generates the data, the relationship between the two parameters is key. In addition, the estimated modes correlate (i.e., preserve rank-order) with each parameter, even if the absolute values are not recovered.

This expression fuses the two forms of performance data generated by the model (Sander and Beyerer, 2013; Wei et al., 2020). The log likelihood of choices is based upon Equation (5). The log likelihood of reaction times is obtained by inverting the expression in Equation 6. In both cases, we have made the dependence upon **c** and **e** explicit. Given this log likelihood, we can employ variational Laplace, as before, to infer the posterior densities for **c** and **e**. Fig. 9 illustrates the mode and 90% credible intervals of these inferences using data generated (using the same stimulus stream) under various values of the **c** and **e** parameters. To assess parameter-recovery, we use simulations generated with a range of different parameter settings. Specifically, we used the parameters in Fig. 8, where the sequence of parameter settings in Fig. 9 correspond to the parameters in the matrix of Fig. 8a, after concatenating the rows. We hoped to see a clear relationship between the parameters used to simulate the data and the recovered parameters.

As shown in Fig. 8, the recovery of the **c** parameter is generally very good, while the **e** parameter is relatively poor (i.e., the estimate is mostly outside of the credible interval) with some improvement in datasets 16–25. Note however, that the rank order of estimates for **e** is preserved (higher ‘true’ values of **e** are associated with higher estimated values). Where the estimates are poor, this implies the model has found an alternative, simpler, way to generate the same data through different combinations of parameters than those that generated the data. However, as shown in the lower panel, the difference between the **c** and **e** parameters is inferred much more reliably. This occurs because there is a high degree of covariance between the two parameters under the posterior estimates, meaning that the marginal density for each parameter neglects an important degree of freedom. The expected differences between the two account for this covariance. The implication is that, in explaining data under the hypothesis that differences in cognitive effort underwrite behavioural differences, it is the motivation to deploy cognitive effort *relative to* the effort demanded by the task (for that individual) that matters, and not the absolute value of the motivation or demand. In principle, this means a single parameter related to effort may be sufficient to characterise performance on the Stroop task. In summary, this parameter recovery exercise demonstrates that it is possible to use active inference to ‘phenotype’ (and infer the prior beliefs of) participants of a Stroop task; and suggests that the most reliable parameter to be used in empirical studies is the difference between the **c** and **e** parameters in our model. This is consistent with previous studies that have shown it is difficult to independently estimate the absolute values of cognitive demand and motivation. For instance, Caplin et al. (2020), in an economic context, focus upon the relationship between analogous parameters via an ‘incentive-based psychometric curve’ as opposed to absolute parameter values, and Musslick et al. (2019) also note the collinearity between demand and motivation. Having said this, our successful recovery of the **c** parameter and the preservation of rank order in estimation of the **e** parameter (analogous to the ordinal relationships achieved by Musslick et al. (2018)) is encouraging that these data do provide information about each parameter independently, and that they may be useful measures in computational phenotyping.

Our final analysis followed on from that shown in Fig. 9. We re-fitted the model with and without each data modality (reaction times and choice data) to establish the relative information afforded by each. Information gain is quantified by the KL-Divergence from our prior beliefs about each parameter to our posterior beliefs following fitting of the model to (simulated) data. This approach has previously been employed for the purposes of feature selection, when the most informative data-features need to be chosen from imaging data to inform neuronal models (Zeidman et al., 2019). Table 1 shows the results of this analysis. The main result is that reaction time data alone provides much more limited information about the **c** and **e** parameters compared to either the combined reaction time and choice data, or the choice data alone. A secondary result is that, for many of the synthetic datasets, more information is provided by the choice data alone than by the combination of choice and reaction time data, despite non-zero information provided by reaction time data alone (although this is not a consistent effect). While counterintuitive, this result depends upon the fact that information gain is not a distance measure, implying we cannot simply add the information gain associated with two data modalities individually and expect to arrive at their combined information gain (Amari and Cichocki, 2010). The key conclusion to draw from Table 1 is that both modalities are informative about our parameters of interest. However, if we had to pick one, choice data is the more useful of the two.

**Table 1:** This table quantifies the information gain, quantified as the KL-Divergence from prior to posterior beliefs, following fitting of the model to different combinations of reaction time and choice data.

Dataset	Information gain (Choice and reaction time data)/nats	Information gain (Choice data)/nats	Information gain (Reaction time data)/nats
1	4.26	14.73	7.28
2	2.41	11.73	5.35
3	2.15	12.49	3.52
4	5.11	19.08	0.47
5	11.15	30.39	0.28
6	4.04	29.57	5.89
7	1.33	17.21	2.55
8	0.69	11.73	2.42
9	2.05	12.49	0.28
10	11.38	27.06	0.73
11	3.89	43.48	0.99
12	3.03	29.57	1.19
13	2.10	20.42	0.28
14	4.18	11.73	1.34
15	8.80	12.49	3.09
16	6.35	53.02	1.43
17	7.68	53.02	0.93
18	6.67	43.48	1.16
19	9.76	29.57	3.70
20	6.79	11.73	2.78
21	14.42	64.72	6.00
22	13.43	53.02	5.04
23	13.72	53.02	4.45
24	13.78	43.48	5.09
25	18.73	29.57	9.19

## 7 Discussion

In the above, we have introduced a formalisation of cognitive effort, and illustrated its face validity through its manifestation in a commonly used neuropsychological task. This application reproduced established phenomena including the congruency sequence effect (Duthoo et al., 2014; Egner, 2007) i.e., that incorrect responses on a Stroop task are more likely for incongruent stimuli immediately following congruent stimuli. Further to this, we have shown that it is possible to draw inferences about the parameters determining effort from behaviour—noting that these inferences reflect the simplest explanation for behaviour and do not necessarily recover the ‘true’ parameters used to generate those data. Nevertheless, these inferences do tell us something about the ‘true’ parameters—specifically, the difference between the **c** and **e** parameters of the model, which can be conceptualised as the motivation to deploy cognitive effort *relative to* the effort demanded by the task (for a particular individual). In principle, this type of inversion could be used to track disease progression or recovery over time (or with alternative treatments), through estimating the parameters that best explain behaviour and their evolution. For instance, this could help in predicting disease trajectories in (frontotemporal) dementias (Matías-Guiu et al., 2019) or in measuring rehabilitation efficacy in traumatic brain injury patients (Ben-David et al., 2011). It may also be helpful in evaluation of psychiatric conditions in which aspects of cognitive control are impaired (Grahek et al., 2019). Quantitative phenotyping of this sort has the benefit of being mechanistic, in the sense that estimated parameter values can be used to generate behaviour characteristic of that phenotype. This may be particularly useful in predicting behavioural consequences of therapeutics designed to target the parameters in question. It is important to note that limited deployment of cognitive effort is not the only plausible explanation for reduced performance in a Stroop task. As an example, clinical conditions involving visual impairment (or higher order visual function) might limit the applicability of this approach with a visual Stroop task. However, the same principles are likely to apply to modified versions of this task, such as the auditory Stroop task which has been deployed in Parkinson's disease (Janssen et al., 2019), a condition associated with visual dysfunction (Weil et al., 2016).

We illustrated that the inversion of a generative model of this sort can be interpreted neurobiologically, both through the anatomy implied by its conditional independencies (Fig. 5), and by the electrophysiological manifestations of belief-updating (Fig. 7). As a consequence, the parameters determining cognitive effort may themselves be interpreted in terms of their physiological roles. Almost invariably, these kinds of parameters (effectively, inverse temperature, precision, or softmax parameters) play the role of synaptic efficacies (Friston, 2017; Kanai et al., 2015; Moran et al., 2013; Parr et al., 2018) that enhance the influence of some neural populations over others. This is significant in that (*i*) the inferential procedures described above offer a way to estimate synaptic function (more precisely, the relative synaptic function associated with the inputs to those neuronal populations that influence policy selection) and (*ii*) it implies that neuromodulatory pharmacotherapies may target these synapses. In clinical neurology, this may be particularly significant, given apathy—which may reflect insufficient effort to meet demand—is a common feature to several different syndromes (Hezemans, 2020). Difficulty in suppressing impulses is also a feature of tic disorders (Rawji et al., 2020) and can be an adverse effect of some of the medications used to treat Parkinsonism (Grall-Bronnec et al., 2018). Identification of the aberrant priors that cause these problems might then help to guide the choice of therapeutic agent.

The key finding—from our parameter-recovery exercise—was that of the two data modalities, choice data furnished more information about the parameters than reaction times. This coheres with previous findings that choice data (as summarised by the accuracy or error rate) vary significantly between patients with frontal lobe lesions and healthy controls, while reaction times do not (Vendrell et al., 1995). While the most useful data modality will depend upon the question being asked—and multimodal data may be the most useful—our analysis can be read as an endorsement of performance measures that take (possibly summarised) choice data into account (Scarpina and Tagini, 2017). One could go further than this and argue that the most useful summaries of a participant's performance are those that contain all the information required to reproduce their behaviour, qualitatively. This is the advantage of a computational phenotype with parameters that play a mechanistic role in generating behaviour. It also allows us to ask ‘what if’ questions, by taking an individual phenotype and modifying a parameter to see how this might influence behaviour. The ability to address these questions has potential in clinical practice, in which we may wish to ask how a patient (whose phenotype we have estimated) might respond to alternative therapies.

In our simulations, **c** and **e** are separate parameters. This raises the question: Does the brain entertain separate estimates of cognitive demand and motivation to deploy cognitive effort, or does it entertain only the motivation relative to the effort demanded by the task? If we take our generative model as a theoretical framework, the two parameters relate to different constructs—the cognitive demand (**e**) and preferences (**c**)—so we might predict that these are encoded separately. As described in Section 3, one hypothesis is that cognitive demand relates to basal ganglia responses and preferences relate to prefrontal cortex responses. Previous theoretical frameworks have also distinguished between the effort that is required and the effort that is deployed to perform a given cognitive task (e.g., see (Richter, 2016)). For example, Kahneman (1973) distinguishes the ‘evaluation of demands on capacity’ as separate from ‘available capacity.’ Under the current framework, the deployed effort depends on preferences, which relates to longstanding ideas that the importance of success (Richter, 2016) or attractiveness of a goal (Brehm and Self, 1989) determines effort—the idea being that, if an individual places high importance on success in a given task, they will be willing to deploy more effort to perform it. The difference between **e** and **c** that is reconstructed in Fig. 9 resonates with the idea that an individual performs a cost-benefit analysis (Croxson, Walton, O'Reilly, Behrens and Rushworth, 2009; Székely and Michael, 2021) to evaluate the benefit they would gain from exerting effort, relative to the amount of effort that is demanded to perform the task successfully.

It is often assumed that effort is dissociable from the accuracy of performing a given task (e.g. (Borghini and Hazan, 2018; Koelewijn et al., 2012),). The current framework is based on the view that effort is different from—but nevertheless contributes to—task performance, and can therefore be inferred from performance, provided that it is within a certain range. Clearly, when performance is at 100%, different levels of effort could plausibly have been exerted to perform at that level, and this would not be distinguishable from accuracy alone. However, it is possible that in some situations reaction times could differentiate different amounts of effort at a particular level of accuracy. Fig. 7 shows that, under this framework, the amplitude of evoked potentials relate to effort, which is compatible with the common use of event-related potentials (Delogu et al., 2019) and pupillometry (Beatty and Lucero-Wagoner, 2000; van der Wel and van Steenbergen, 2018) to index effort.

Finally, as we have highlighted the compatibility between our approach and those of other authors, it is important to identify where we differ and why. To address this, we first take a specific example, and then consider more general differences. The specific example is drawn from Butz (2022) and is of special relevance as the Resourceful Event-Predictive Inference (REPI) perspective on cognitive effort in that paper also draws upon ideas from active inference. The REPI model has been successful in reproducing some features of effortful behaviour—focusing upon the Simon effect. The model equates cognitive effort with a mutual information between causes and their consequences. Interestingly, this same mutual information appears in the expected free energy:

(10) $$G_{\pi }=o_{\pi \tau }\cdot C+o_{\pi \tau }\cdot ln\;o_{\pi \tau }+H\cdot s_{\pi \tau }=o_{\pi \tau }\cdot C-H\left[Q\left(o_{\tau }|\pi \right)\right]+E_{Q}\left[H\left[P\left(o_{\tau }|s_{\tau }\right)\right]\right]=o_{\pi \tau }\cdot C-\underset{\text{Mutual}\;\text{information}}{\underbrace{D_{KL}\left[Q\left(o_{\tau }|\pi \right)Q\left(s_{\tau }|\pi \right)\|Q\left(o_{\tau },s_{\tau }|\pi \right)\right]}}$$

See, for comparison, Equation (3) in Butz (2022). As a large (negative) expected free energy causes a greater deviation from the habitual prior distribution over policies, a large mutual information means greater deployment of cognitive effort—and a greater chance of overcoming a cognitive habit. In this sense, our formulation is (qualitatively, if not quantitatively) aligned with that of the REPI model.

However, the two formulations differ in relation to the role of preferences. As is evident from Equation 6, when elements of **C**, determining the preferences, are large they can also facilitate deployment of cognitive effort. This means effort may be motivated either by the potential to explore or to exploit. An important aspect of the REPI model—that we have not considered here—relates to the concept of task-switching. Specifically, priors like **E** do not need to be fixed and can themselves be learned or can depend upon higher levels of a deep generative model. Although this deep contextualisation of habitual priors was not necessary for the Stroop task, the machinery to do so, using active inference, has been developed—see, for example (Parr et al., 2021; Parr and Pezzulo, 2021)—and may be necessary for models of effort in the setting of task-switching as addressed by REPI.

More generally, perhaps the most significant conceptual departure from most treatments is that everything in our account is formulated in terms of beliefs. By beliefs, we do not mean consciously held (i.e., propositional) beliefs but probability distributions, which may implicitly be represented by the activities of neuronal populations, and which are updated during perceptual inference. To those unfamiliar with this style of computational neuroscience, it may seem unusual to frame everything in inferential terms. However, the benefit is simplification. There is one process in play; namely, the minimisation of variational free energy through belief-updating. This process is not specific to the Stroop task and has been shown to be applicable to a wide range of tasks and behaviours e.g., (Adams et al., 2015; Brown et al., 2013; Cullen et al., 2018; Daucé and Perrinet, 2020; FitzGerald et al., 2015; Kaplan and Friston, 2018; Pezzulo et al., 2015; Smith et al., 2019; Tschantz et al., 2021). This means the only assumption that must be made is about the form of the forward model people might use to predict sensory outcomes while performing a Stroop task. In proposing this model, all we have done is set out the minimal set of states required to generate stimuli (and expected responses) in a Stroop task. This contrasts with other approaches, which require assumptions about the imposition of ‘top-down attentional biasing’ and other such cognitive processes that might be in play during the task. Rather than make assumptions about the cognitive processes required to solve the task, we simply apply a generic optimisation procedure to a description of the task (the generative model) and ask whether phenomena that look like top-down attentional biasing, conflict monitoring (Botvinick et al., 2001), and the behavioural consequences of effort emerge from inversion of the model. It may be that the computational mechanics ultimately look very similar under different approaches. If so, it is encouraging that different routes to solving the problem arrive at the same destination.

## 8 Conclusion

In summary, we have set out a theory of cognitive effort inspired by information theoretic formulations of this notion (Zénon et al., 2019). This sees effort as the divergence between our beliefs about covert action given only habits and given a full prior belief that accounts for explorative and exploitative drives. In other words, effort is deployed to overcome a mental habit.^5^ Numerical analysis of this formulation showed its ability to influence performance in a common neuropsychological task: a Stroop task. In addition to reproducing the basic Stroop effect, our simulations also produced behaviour consistent with the established congruency sequence effect and the speed-accuracy trade-off that is ubiquitous in the cognitive control literature. We additionally found that, consistent with empirical findings (Manohar et al., 2015), the speed-accuracy trade-off was attenuated with greater preference for being correct. Through our simulations, we observed a clear relationship between priors and behaviour. We show that prior beliefs may be estimated from behaviour using Bayesian inference to overcome the inherent inverse problem. This implies simple behavioural tasks may be sufficient to phenotype those with heterogenous cognitive syndromes according to the balance between cognitive demand and effort. A key finding of this work is that behavioural choice data appears to be more informative than reaction times, although both contribute useful information, in characterising the parameters that underwrite performance of a Stroop task. An important reason for wishing to estimate these phenotypic parameters is that they enable predictions about the effort individual participants might experience when performing a Stroop task. This offers an opportunity to evaluate the validity of our proposed definition of effort by comparing these predictions with the subjective experiences of experimental participants.

## Author contribution statement

All authors contributed directly and substantially to this paper.

## CRediT author statement

Thomas Parr: Conceptualization; Methodology; Software; Formal analysis; Writing - Original Draft; Visualization; Emma Holmes: Conceptualization; Writing - Review & Editing; Karl Friston: Conceptualization; Software; Resources; Writing - Review & Editing; Giovanni Pezzulo: Conceptualization; Writing - Review & Editing.

## Software availability

The Matlab routines used to generate these simulations (spm_MDP_VB_X.m) are freely available as part of the SPM 12 package available at https://www.fil.ion.ucl.ac.uk/spm/. The specific figures from this paper can be reproduced using the DEMO_MDP_Stroop.m routine which will be available in the next public SPM release or on request from the corresponding author.

## Declaration of competing interest

The authors declare that they have no known competing financial interests or personal relationships that could have appeared to influence the work reported in this paper.

## Data Availability

No data was used for the research described in the article.

## Footnotes

1 We use the terms ‘mental’ and ‘cognitive’ interchangeably when referring to effort or habit.

2 As **G** is a vector of potentials (i.e., unnormalized negative log probabilities), the condition **G** = **0** is equivalent to any **G** in which all elements take the same value.

3 For the avoidance of confusion, the states of the generative model are descriptions of the structure of the task. Sometimes the term ‘task set’ is used to refer to the cognitive processes in play when engaging in that task (Sakai, 2008). From our perspective, this use of the term corresponds to the process of forming beliefs about the instruction state.

4 Log scale parameters are a common device in statistical inference to ensure positivity of the estimated parameter.

5 Zénon et al. (2019) suggest a broader formulation of effort, in which effort can be linked to any attempt to overcome a prior belief; be it about policies or something else.
